# Editorial: Woody plants and climate resilience: responses to environmental stress and weather extremes

**DOI:** 10.3389/fpls.2026.1945018

**Published:** 2026-08-14

**Authors:** David W. M. Leung, Rambod Abiri

**Affiliations:** 1 School of Biological Sciences, University of Canterbury, Christchurch, New Zealand; 2 Department of Plant Agriculture, University of Guelph, Guelph, ON, Canada

**Keywords:** irrigation, metabolome, plant phenotyping, tree mortality, WOX transcription factors

Stressful environmental conditions and extreme weather such as drought, and temperature fluctuations in the contemporary context of climate change are expected to impact upon forests, agroforestry systems, commercial tree orchards and urban landscapes. Woody plant growth and productivity, ecosystem stability, biodiversity, and carbon cycling are vulnerable to these adverse environmental conditions. In this Research Topic, the studies aimed to obtain a better understanding of the short- and long-term resilience mechanisms of woody plants to diverse environmental stressful conditions. This knowledge resource is critical in developing effective strategies to protect woody plant populations and ensure their survival under increasingly harsh environmental conditions as well as providing a theoretical foundation for breeding of more abiotic stress resilient commercial tree varieties.

Lee et al. approached tree mortality problem at the stand level rather than individual tree mortality as multiple tree mortality could better reflect the impact of climate change and environmental stress factors. A key knowledge gap in multiple tree mortality and stand regeneration was addressed by studying, in the same timeframe, both the aboveground changes such as shifts in species composition and the belowground changes in microbial communities in *Pinus densiflora* forests. DNA extracted from rhizosphere soil samples was sequenced and bioinformatic analysis was performed. This study highlighted the significance of the interactive dynamic of aboveground vegetation changes and belowground indicator rhizosphere microbial community in forest regeneration.

Dito et al. investigated how to improve irrigation management in tree orchards, particularly hazelnut orchards. While irrigation is increasingly used in tree orchards all over the world as temperature rises in summer in recent years, excessive irrigation could cause root zone flooding resulting in reduced growth and productivity loss. In this study, the relationship between midday stem water potential and vapor pressure deficit affecting stomatal conductance was explored to collect data to establish a baseline that can be applied in the field to know when over irrigation occurs. This contributes to the overarching goal of sustainable use of the limited water resources under fluctuating climatic conditions.

Daniele et al. evaluated non-invasive ornamental shrubs as alternatives to invasive non-native plants in urban settings, particularly in European cities where two dominant abiotic stressors, drought and salinity could reduce plant growth. *Ligustrum sinense* (an invasive plant), and two non-invasive *L. japonicum* ‘Texanum’, and *L. vulgare* that could provide multiple ecological services were grown in a growth chamber under controlled experimental conditions. The 3-dimensional (3D) morpho-physiological characteristics of the plants in response to drought and salinity stress applied separately and in combination were obtained using an advanced, automated plant phenotyping platform with a single-line laser, sensors and multispectral cameras. This approach enabled a more detailed comparison than 2D methods of monitoring the adaptation traits of potential alternative plants to invasive plants for urban settings under abiotic stress conditions.

Ndayambaza et al. examined genome-wide the WUSCHEL-related homeobox (WOX) transcription factors in *Populus euphratica*, a model drought and salinity resilient woody plant adapted to harsh desert conditions in the Northwest of China. While WOX transcription factors are known to be involved in plant development and stress resilience in crops and trees, there is a lack of more comprehensive understanding of the relationship between WOX genes in regulation of plant development and abiotic stress resistance in trees. In this study, 18 WOX genes were identified and characterized in the genome of *P. euphratica*, and as expected differential expression patterns of some of these WOX genes were found in response to prolonged exposure to drought and salt stress. More importantly, it was also found that some WOX genes appear to be involved in fine tuning shoot, leaf and root development adapted to abiotic stress.

Luo et al. sought to elucidate the key determinants of heat stress tolerance in mango leaves of a heat stress tolerant cultivar in comparison with two less heat tolerant cultivars. This is a world first study of combining metabolomic and transcriptomic approaches to study acute heat stress response to 40 ^0^C for up to 8 h. It was found that 12 major metabolite classes including amino acids and phenolic acids were most responsive to heat stress. Based on RNA-seq analysis coupled with powerful statistical analysis including integrated Pearson correlation analysis, it was possible to uncover a network of genes and metabolites involved in early heat stress response. This study would be valuable to future work for breeding heat tolerant mango varieties.

The studies in this Research Topic investigated the diverse climatic challenges including drought, salinity and heat stress affecting woody plants in natural forest, urban and commercial orchard environments. In these studies, contemporary approaches including establishing a field-applicable physiological baseline to avoid the problem of over irrigation, automatic plant phenotyping, rhizosphere microbial DNA sequencing, genome-wild gene analysis, metabolomic and transcriptomic analyzes were applied. Some foundation knowledge for strategies to safeguard the future of woody plants under increasingly harsh environmental conditions has been obtained. More further studies will add to this knowledge base of resilience mechanisms in woody plants to cope with climatic extremes.

